# A positive Real-Time Elastography (RTE) combined with a Prostate Cancer Gene 3 (PCA3) score above 35 convey a high probability of intermediate- or high-risk prostate cancer in patient admitted for primary prostate biopsy

**DOI:** 10.1186/s12894-016-0159-1

**Published:** 2016-07-08

**Authors:** Yngve Nygård, Svein A. Haukaas, Ole J. Halvorsen, Karsten Gravdal, Jannicke Frugård, Lars A. Akslen, Christian Beisland

**Affiliations:** Department of Urology, Haukeland University Hospital, N-5021 Bergen, Norway; Department of Pathology, Haukeland University Hospital, N-5021 Bergen, Norway; Department of Clinical Medicine, University of Bergen, Bergen, Norway; Center for Cancer Biomarkers CCBIO, Department of Clinical Medicine, University of Bergen, Bergen, Norway

**Keywords:** Prostate cancer, Ultrasound, Diagnosis, PCA3, Real-time elastography, RTE

## Abstract

**Background:**

The standard of care in patients with suspected prostate cancer (PCa) is systematic prostate biopsies. This approach leads to unnecessary biopsies in patients without PCa and also to the detection of clinical insignificant PCa. Better tools are wanted. We have evaluated the performance of real-time elastography (RTE) combined with prostate cancer gene 3 (PCA3) in an initial biopsy setting with the goal of better identifying patients in need of prostate biopsies.

**Methods:**

127 patients were included in this study; three were excluded because of not measureable PCA3 score leading to 124 evaluable patients. A cut-off value of 35 was used for PCA3. All patients were examined with a Hitachi Preirus with an endfire probe for RTE, a maximum of five targeted biopsies were obtained from suspicious lesions detected by RTE. All patients then had a 10-core systematic biopsy performed by another urologist unaware of the RTE results. The study includes follow-up data for a minimum of three years; all available histopathological data are included in the analysis.

**Results:**

There was a significant difference in PCA3 score: 26.6 for benign disease, 73.6 for cancer patients (*p* < 0.001). 70 patients (56 %) were diagnosed with prostate cancer in the study period, 21 (30 %) low-risk, 32 (46 %) intermediate-risk and 17 (24 %) high-risk. RTE and PCA3 were significant markers for predicting intermediate- and high-risk PCa (*p* = 0.001). The combination of RTE and PCA3 had a sensitivity of 96 % and a negative predictive value (NPV) of 90 % for the group of intermediate- and high-risk PCa together and a NPV for high-risk PCa of 100 %. If both parameters are positive there is a high probability of detecting intermediate- or high-risk PCa, if both parameters are negative there is only a small chance of missing prostate cancer with documented treatment benefit.

**Conclusions:**

RTE and PCA3 may be used as pre-biopsy examinations to reduce the number of prostate biopsies.

## Background

The mainstay in the diagnosis of prostate cancer (PCa) is biopsy-driven by serum prostate-specific antigen (PSA) and digital rectal examination (DRE). There is really no level of PSA that excludes PCa, and many benign prostatic diseases may cause PSA elevation. The threshold value of PSA for prostate biopsy is arbitrarily chosen, which is dependent on the age of the patient, life expectancy and the size of the prostate. It is well recognized that PSA screening results in both the over-diagnosis and overtreatment of prostate cancer [1–3]. Furthermore, a lot of men with benign disease are going through prostate biopsy without any beneficial effects. There is also an increase in biopsy-related infections because of antibiotic resistant bacteria, and some of these infections can be lethal [4, 5]. There is a need to better identify those men not harboring PCa to avoid unnecessary biopsies and related complications.

Currently, there is little enthusiasm for population-based PSA screening, and in May 2012 the U.S. Preventive Services Task Force recommended against routine PSA screening [6]. Moreover, European Association of Urology (EAU) Guidelines (2013) do not support programmed mass PSA screening, while recommending early detection in well-informed men [7].

To assist in the decision to perform prostate biopsy, nomograms have been created. The US Food and Drug Administration has approved prostate cancer gene 3 (PCA3) as a predictive test prior to performing a repeat biopsy. PCA3 has shown to enhance the performance of nomograms based on initial biopsy results [8, 9].

Standard systematic prostate biopsy is performed by placing a biopsy needle in 10 to 12 prostate sectors of the peripheral zone under transrectal ultrasound (US) guidance. Cancer in the central or anterior part of the prostate may be overlooked, and insignificant cancer detected with such biopsy regimens [10].

Imaging techniques, specifically advanced US and multiparametric MRI (mpMRI), are evolving, and thereby making it possible to identify areas suspected of harboring PCa [11, 12]. Targeted biopsy guided by RTE detects high-grade cancer, although it misses some significant cancers compared with a systematic 10-core biopsy [13, 14]. mpMRI, together with fusion into real-time US, is practical for targeted biopsy but this approach also misses significant PCa [15].

In a prospective series of patients undergoing radical prostatectomy, the combination of RTE and PCA3 detected 97 % of significant PCa [16]. The present study was undertaken to evaluate prospectively the capability of RTE and PCA 3 to predict clinically significant PCa in patients admitted for initial prostate biopsy.

## Methods

The study was carried out in the outpatient clinic of the Department of Urology at Haukeland University Hospital from February 2011 to June 2012. The Regional Committee for Medical and Health Research Ethics in Western Norway approved the study.

A total of 127 consecutive patients were included using active inclusion, with only a very small amount of patients declining to participate. The inclusion criteria were a PSA level 3 – 25 ng/ml, age ≤75 years and no prior biopsies within the last five years, in addition to the patients being amenable for radical treatment.

At first, DRE was performed in all patients to determine clinical stage (cT) and to perform the prostatic massage needed before urine sampling. Before further evaluation, the first stray urine was captured and transferred to the transportation tubes needed for the PCA3 analysis. We used Progensa™ PCA3 analysis, and the tests were analyzed at the Fürst Medical Laboratory in Oslo, Norway. After the urine test, all patients were given a single dose of Ciprofloxacin 1 g as an antibiotic prophylaxis. All patients were examined in the left decubital position, with the ultrasound procedures being thoroughly previously described [16]. In brief, all patients were examined using a Hitachi Preirus Ultrasound machine with software for RTE. They were first examined using a V53W transrectal end-fire probe for B-mode evaluation, determination of prostate volume (Pvol), RTE and targeted biopsies. The peripheral zone (PZ) of the prostate was divided in six region of interest (ROI), one at the base, one at the mid prostate and one at the apex on each side. All RTE-reproducible hard lesions of more than 5 mm were allocated to the corresponding ROI. Furthermore, two to four targeted biopsies were taken from suspicious ROIs. A CC531 transrectal simultaneous biplane probe was used for standard systematic biopsies. In the same setting a different urologist blinded for the RTE results performed a 10-core systematic biopsy from the six ROIs. The biopsies were fixated in formaldehyde and analyzed by two uro-pathologists. Total core length, as well as the length of cancer tissue and Gleason grade and score, was separately recorded for each biopsy core.

**Fig. 1:**
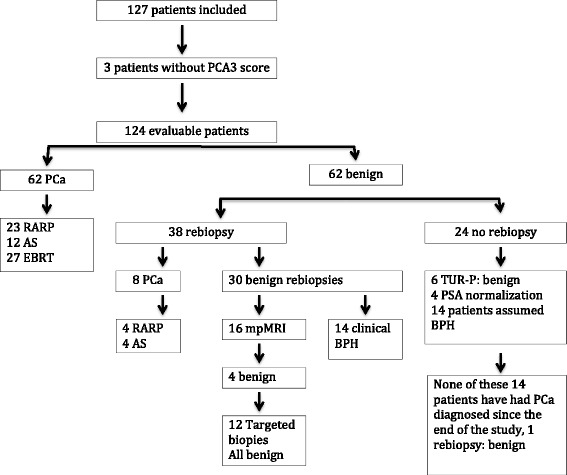
Flowchart of the 127 included patients in this study. The numbers indicate the number of patients in each group. Abbreviations: PCA3: Prostate cancer gene 3; PCa: Prostate cancer; RARP: Robotic assisted radical prostatectomy; AS: Active surveillance; EBRT: External beam radiation therapy; TUR-P: Transurethral resection of prostate; PSA: Prostate specific antigen

In the statistical analyses, we included not only the results and outcomes of the initial biopsy, but also at least three years of follow-up data for the patients. If there was a clinically persisting suspicion of PCa after the initial biopsies, patients were monitored closely (see Fig. 1). Repeat biopsies were performed in 38 patients within the next six months, while in 24 patients no repeat biopsy was performed. Sixteen patients with benign repeat biopsies went through a mpMRI of the prostate, and in 12 of these we performed targeted biopsies of suspicious areas by TRUS guided biopsies with a “cognitive fusion” of mpMRI. Together with an uro-radiologist, a trained urologist performed such biopsies. All biopsy data were included in the analyses. Among those 24 patients with no repeat biopsy, four patients experienced a normalization of PSA levels at follow-up, six were admitted for TUR-P with a benign pathology, and in 14 patients benign prostatic hyperplasia was assumed as the reason for a slight elevation in PSA level. The medical records for these patients and the registry at our department of pathology were examined in October 2015 to identify whether PCa had been diagnosed since the end of inclusion. The mean observation time for these patients is 46.7 ± 1.5 months (median 44.4, range 41–55). The medical records for the 14 patients with benign repeat biopsies were also examined at the same time, though none have had PCa diagnosed in this period.

### Statistical analyses

Standard descriptive statistics were used and presented as mean and median. A 95 % confidence interval (CI) was calculated. Negative predictive value (NPV), positive predictive value (PPV), sensitivity and specificity were calculated for RTE by ROI and by patient, for PCA3 using a cut-off value of 35 and for a combination of both. Different groups were compared using the exact Chi-square test, a Mann–Whitney U-test and the t-test for categorical, ordinal and continuous data, respectively. A multiple logistic regression model was estimated entering the clinical parameters age, Pvol and PSA alone, or combined with a dichotomized PCA3 score of 35 and positive RTE by patient. DRE is commonly used in such clinical models but we excluded DRE from the model because DRE and RTE both are parameters expressing tissue stiffness. The performance of the calculations was expressed as the area under the curve (AUC) of the receiver operating curves (ROC). A 95 %CI was calculated for the AUC and displayed in parenthesis after AUC.

## Results

In three patients the urine did not contain enough cells for the PCA3 analysis resulting in 124 evaluable patients.

A total of 70 (56 %) patients were diagnosed with PCa, of whom 62 were identified in the initial biopsy setting and eight patients at the repeat biopsy. The inclusion of these eight patients did not alter the diagnostic performance of RTE by ROI as the sensitivity, specificity, PPV and NPV were 43, 84, 49 and 80 %, respectively; the false positive rate was 16 % and the false negative rate 12 %.

According to the European Association of Urology (EAU) risk stratification, there were 21 (30 %) low-, 32 (46 %) intermediate- and 17 (24 %) high-risk cancers [7]. In the eight patients detected with PCa on the repeat biopsies six were low-risk and two were intermediate-risk cancers, there were no high-risk PCa in this group.

The distribution of PSA, PCA3 score, Pvol, age and proportion of positive DRE for all patients and for patients with and without PCa is found in Table 1. The p-values are calculated for the difference between the groups with PCa and without PCa. The clinical stage, biopsy Gleason grade and score and risk stratification according to EAU guidelines are also detailed in Table 1.

**Table 1:** Patient characteristics of 124 patients of whom 70 were diagnosed with PCa

Variable		Total (*n* = 124)	PCa (*n* = 70)	No PCa (*n* = 54)	*p*-value*
PSA	Mean (Median; 95 %CI)	9.1 (7.2; 8.3-9.9)	9.7 (7.7; 8.5-11.0)	8.3 (6.7; 7.2-9.4)	0.090*
PCA3-score	Mean (Median; 95 %CI)	53.1 (33.5; 42.9-63.4)	73.6 (53.5; 57.7-89.6)	26.6 (19.0; 19.9-33-2)	<0.001*
Prostate volume	Mean (Median; 95 %CI)	60.0 (53.0; 54.7-65.4)	49.9 (43.5; 44.7-55.1)	73.2 (66.5; 63.8-82.5)	<0.001*
Age	Mean (Median; 95 %CI)	64.0 (65.1; 62.9-65.2)	64.9 (65.7; 63.5-66.2)	62.9 (63.0; 61.0-64.9)	0.094*
Positive DRE	Number (%)	31 (25 %)	22 (31 %)	9 (17 %)	0,060**
Clinical stage	Number (%)				
T1c			35 (50 %)		
T2a			12 (17 %)		
T2b			6 (9 %)		
T2c			11 (16 %)		
T3a			6 (9 %)		
Gleason score	Number (%)				
3 + 2 = 5			1 (1 %)		
3 + 3 = 6			21 (26 %)		
3 + 4 = 7a			15 (19 %)		
4 + 3 = 7b			9 (11 %)		
4 + 4 = 8			5 (6 %)		
4 + 5 = 9			4 (5 %)		
5 + 4 = 9			2 (2 %)		
EAU-risk	Number (%)				
Low-risk			21 (30 %)		
Intermediate-risk			32 (46 %)		
High-risk			17 (24 %)		

*PCa* prostate cancer, *PSA* prostate specific antigen, *PCA3* prostate cancer gene 3, *DRE* digital rectal examination is considered positive if there was suspicion of PCa

*p-value is estimated for the difference of means between the group with PCa and the group without PCa using the t-test

**p-value is estimated for the difference of proportions between the group with PCa and the group without PCa using Chi-square test

RTE was positive in 85 cases and negative in 39 . The average PCA3 score in patients with PCa was significantly higher compared with normal or benign disease (73.6 vs. 26.6, *p* < 0.001). For PSA, there were no statistical significant differences between those patients with- and those without PCa (9.7 vs. 8.3, *p* = 0.09).

The sensitivity, specificity, PPV and NPV of RTE by patient, PCA3 at score 35 and the combination of both for any PCa, for intermediate- and high-risk PCa together, and for high-risk PCa alone, are shown in Table 2.

**Table 2:** This table shows the diagnostic performance of RTE and PCA3 score with cut-off 35 for the group of any PCa, for the combined group of intermediate-and high-risk PCa, and for high-risk PCa

	Parameter	Sensitivity	Specificity	NPV	PPV
Any PCa	RTE	74 %	39 %	54 %	61 %
	PCA3	64 %	78 %	66 %	80 %
	Combination	91 %	26 %	70 %	62 %
IR and HR PCa	RTE	86 %	43 %	82 %	51 %
	PCA3	71 %	66 %	78 %	58 %
	Combination	96 %	24 %	90 %	55 %
HR PCa	RTE	88 %	35 %	95 %	18 %
	PCA3	82 %	57 %	95 %	23 %
	Combination	100 %	19 %	100 %	16 %

*Abbreviations*: *PCa* prostate cancer, *IR* intermediate-risk, *HR* high-risk, *RTE* real-time elastography, *PCA3* prostate cancer gene 3, *NPV* negative predictive value, *PPV* positive predictive value

In univariate logistic regression analysis a positive RTE was a highly significant predictor of intermediate- risk and high-risk PCa (Table 3).

**Table 3:** Logistic regression analyses for predicting high and intermediate risk prostate cancer (*n* = 124)

	Simple			Multiple					
	Unadjusted			Fully adjusted			Final model		
Variables	OR	95 % CI	*p*-value**	OR	95 % CI	*p*-value**	OR	95 % CI	*p*-value**
Age (cont. in years)	1.04	(0.98,1.10)	0.188	1.04	(0.96, 1.13)	0.287			
PSA (cont. in ng/ml)	1.18	(1.08, 1.29)	<0.001	1.19	(1.07, 1.34)	0.001	1.18	(1.03, 1.14)	0.001
Pvol. (cont. in ml)	0.98	(0.96, 0.99)	0.003	0.97	(0.95, 0.99)	0.005	1.04	(1.04, 1.07)	0.009
Positive RTE (Y/N)	4.46	(1.78, 11.22)	0.001	2.73	(0.96, 7.79)	0.052	2.56	(0.91, 7.23)	0.068
PCA3 (>35 vs. <35)	5.00	(2.28, 10.95)	<0.001	3.31	(1.27, 8.63)	0.013	4.12	(1.71, 9.91)	0.001

*Abbreviations*: *RTE*: real-time elastography, *Cont* continuous, *Y/N* yes/no, *OR* odds ratio, *CI* confidence interval, *Pvol* prostate volume

**p-value by the use of the Likelihood Ratio test

Entering PCA3 and RTE in a clinical model encompassing age, PSA and Pvol; PSA, Pvol and PCA3 were independent predictors of intermediate-risk and high-risk PCa while RTE showed a tendency toward significance (Table 3).

**Fig. 2:**
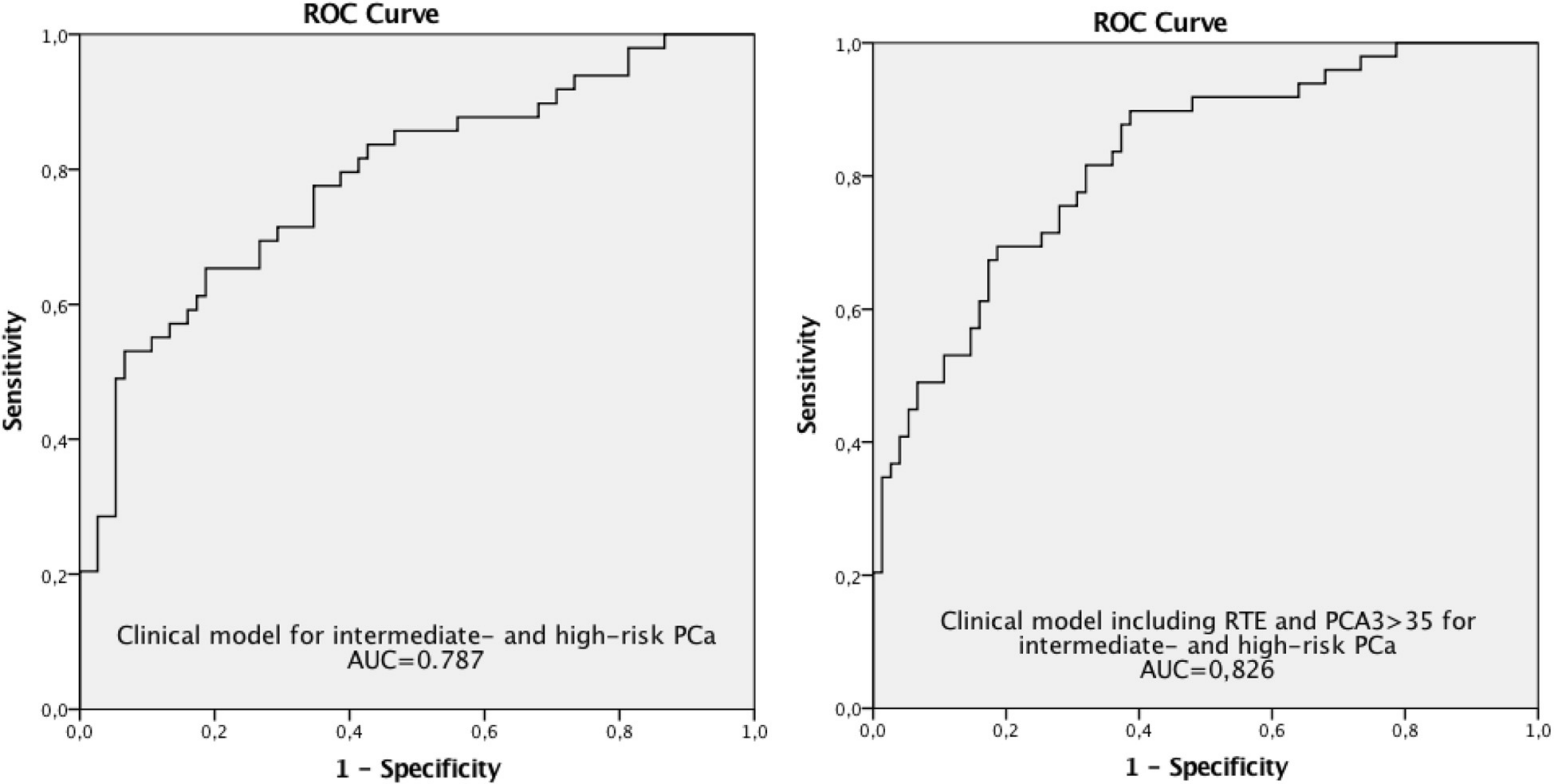
ROC curves for the regression analyses for the group of intermediate- and high-risk PCa. The addition of PCA3 score >35 and RTE lead to an increase in AUC of 0.039. Abbreviations: PCa = Prostate cancer, ROC = receiver operating curves, PCA3 = prostate cancer gene 3, AUC = area under curve

The results of the logistic regression analyses were also expressed in a ROC curve that yielded an AUC of 0.826 (0.752-0.899) for the complete model and 0.787 (0.703-0.872) for the clinical model alone (Fig. 2).

To evaluate the clinical impact of the combination of PCA3 and RTE, we utilized the most commonly used cut-off value of 35 for PCA3, and allocated the patients into four groups.

Group 1 included patients for whom both RTE and PCA3 were positive. Patients with a positive RTE and negative PCA3 were put into Group 2, and RTE negative and PCA3 positive patients were allocated to Group 3. Finally, Group 4 encompassed patients negative for RTE, as well as PCA3. Group 1 encompassed 44 patients; 30 had a high- or intermediate-risk PCa, eight a low-risk PCa and six a benign prostate. If both tests were positive, we found a high (86 %) probability of PCa at biopsy. On the other hand, of 23 patients with a PCA3 below 35 and a negative RTE (Group 4), eight patients were diagnosed with PCa, including six with low-risk cancer and two with intermediate-risk cancer, while 15 patients did not have any cancer. There was no high-risk PCa in this group. Omitting a biopsy in this group would imply a 9 % likelihood of missing PCa of clinical importance. In Group 2, 14 patients were diagnosed with cancer and 27 without cancer. There were 16 patients with a PCA3 score equal to or higher than 35 and a negative RTE (Group 3); ten patients had cancer and no cancer was found in the other six. The results achieved from pre-biopsy PCA3 urinary tests and RTE assessments in both Group 1 and Group 4 are informative and may be of benefit in the decision-making process as to whether to perform a biopsy or not.

Out of 70 patients for whom PCa was diagnosed, 27 underwent radical prostatectomy, 27 received external radiotherapy and 16 opted for active surveillance.

## Discussion

There is a changing wind in the way we detect and treat PCa as a consequence of the well-known over-diagnosis and overtreatment of PCa, in addition to the documented increasing rate of post-biopsy infections [4, 5]. There is an ongoing search for new biomarkers and the development of improved methods for identifying clinically significant PCa. Evolving evidences show the benefit of PCA3 in the decision-making process of performing repeat biopsies in men where the initial biopsy is negative.

Both RTE and mpMRI are capable of identifying PCa that is not visualized on B-mode ultrasound [17, 18].

To the best of our knowledge, the present paper is the first to present prospective data on the combination of pre-biopsy PCA3 and RTE by patient in predicting PCa in an unselected series of men admitted for an initial biopsy.

The most important findings are the high sensitivity as well as NPV in predicting intermediate-risk and high-risk PCa (Table 2). PCA3 and RTE appeared to be of benefit mostly in patients if both parameters were positive or negative. If both parameters are positive, there is good reason to perform a biopsy and there is a high probability of detecting aggressive disease. Additionally, avoiding a biopsy in which PCA3 and RTE are negative carries a small risk of missing patients harboring a clinically significant PCa. In this series we found 32 intermediate-risk PCa and 17 high-risk PCa. By using RTE and PCA3 as selection criteria for performing a biopsy, 23 patients would have been advised against having a biopsy; only two of these patients had intermediate-risk PCa and no patients had high-risk PCa. One could argue that the reduction of unnecessary biopsies is relatively small since only 23 patients (19 %) would have been advised against biopsy. On the other hand, these patients could safely be advised against biopsy, as every reduction of unnecessary biopsies is a step in the right direction in reducing over-diagnosis and overtreatment of low-risk PCa. These findings are in line with our previous study of the combination of PCA3 and RTE in a smaller series of radically operated PCa patients [16].

In the logistic regression analysis PCA3 as well as a positive RTE contributed to the clinical model although RTE achieved a p-value close to significance (0. 068). In a ROC analysis, the full model with PCA3 and RTE achieved an AUC of 0.826. In univariate analysis a positive RTE is a highly significant predictor of PCa.

No definite threshold of PCA3 score has been agreed upon as yet, although a score of 35 is most frequently used as a cut-off value. In our study, we tested two different PCA3 score thresholds of 21 and 35, respectively. A threshold score of 35 provided the most optimal PPV of 80 %, which is the same figure found in a prospective randomized study by Wei et al., using a PCA3 score threshold of 60 in the initial biopsy setting [19]. In our analyses, we utilized a PCA3 score of 35 as the threshold value.

A strength of this study is that it includes histo-pathological data on initial biopsies, repeat biopsies as well as data of further follow-up, including mpMRI targeted biopsies of suspected lesions. No patients in the group diagnosed with a benign disease have been diagnosed with PCa in the period since the study inclusion was closed in June 2012. For all 14 patients with a presumably benign reason for an elevated PSA, both medical records and records for the regional pathology laboratory were checked. We believe that we are as close as possible to the true prevalence of PCa in the study population at the time of the examinations. This makes this study different from other studies investigating PCA3 [8, 20] and RTE [21, 22], in which the performance of these markers has been solely evaluated at the initial biopsy.

In this series of patients, a total of 70 patients were diagnosed with PCa, including 21 who were classified as low-risk and 49 as either high- or intermediate-risk.

Analyzing the group of PCa patients harboring either high- or intermediate-risk PCa, the combination of RTE and PCA3 correctly identified 47 of these patients. That means we correctly identified 96 % of the patients harboring PCa in need of treatment in a pre-biopsy setting. This result may be used to reduce the number of unnecessary biopsies at a small risk of missing PCa in need of treatment.

The present study has some limitations. Firstly, it is a single center, single investigator study. RTE like all US investigations are real-time examinations and are operator dependent and an inter-observer investigation would have been of value. As to the learning curve, it has been shown that after about 30 RTE the novice is achieving comparable results to experienced US operators [23]. Secondly, a relatively small number of patients are included. Thirdly, there is a limited number of patients with high-risk PCa, although the findings are in line with our previously published paper on patients planned for radical prostatectomy [16].

## Conclusions

In patients with a positive RTE combined with a PCA3 score above 35 there is a high probability of detecting intermediate- or high-risk PCa. The combination of these markers correctly identified 47 of 49 (96 %) patients in need of a further diagnostic work-up. The high NPV of the combination of PCA3 and RTE makes it possible to avoid some 20 % of the prostate biopsies without missing high-risk PCa. If applied to the upper age group, in which a missing low-risk PCa may be seen as an advantage, the use of RTE and PCA3 may be implemented as pre-biopsy examinations to reduce the number of prostate biopsies.

## Abbreviations

AUC, area under the curve; CI, confidence interval; DRE, digital rectal examination; EAU, European Association of Urology; mpMRI, multiparametric magnetic resonance imaging; NPV, negative predictive value; PCa, prostate cancer; PCA3, Prostate cancer gene 3; PPV, positive predictive value; PSA, prostate-specific antigen; Pvol, prostate volume; ROC, receiver operating curve; RTE, real-time elastography; TRUS, transrectal ultrasound; US, ultrasound

## References

[1] Ilic D, Neuberger MM, Djulbegovic M (2013). Screening for prostate cancer. Cochrane Database Syst Rev.

[2] Chou R, Croswell JM, Dana T (2011). Screening for prostate cancer: a review of the evidence for the U.S. Preventive Services Task Force. Ann Intern Med.

[3] Schroder FH, Hugosson J, Roobol MJ (2014). Screening and prostate cancer mortality: results of the European Randomised Study of Screening for Prostate Cancer (ERSPC) at 13 years of follow-up. Lancet.

[4] Carignan A, Roussy JF, Lapointe V (2012). Increasing risk of infectious complications after transrectal ultrasound-guided prostate biopsies: time to reassess antimicrobial prophylaxis?. Eur Urol.

[5] Loeb S, Vellekoop A, Ahmed HU (2013). Systematic review of complications of prostate biopsy. Eur Urol.

[6] Moyer VA (2012). Force USPST Screening for prostate cancer: U.S. Preventive Services Task Force recommendation statement. Ann Intern Med.

[7] Heidenreich A, Bastian PJ, Bellmunt J (2014). EAU guidelines on prostate cancer. part 1: screening, diagnosis, and local treatment with curative intent-update 2013. Eur Urol.

[8] Hansen J, Auprich M, Ahyai SA (2013). Initial prostate biopsy: development and internal validation of a biopsy-specific nomogram based on the prostate cancer antigen 3 assay. Eur Urol.

[9] Nygard Y, Haukaas SA, Eide GE (2015). Prostate cancer antigen-3 (PCA3) and PCA3-based nomograms in the diagnosis of prostate cancer: an external validation of Hansen's nomogram on a Norwegian cohort. Scand J Urol.

[10] Haas GP, Delongchamps NB, Jones RF (2007). Needle biopsies on autopsy prostates: sensitivity of cancer detection based on true prevalence. J Natl Cancer Inst.

[11] Walz J, Marcy M, Pianna JT (2011). Identification of the prostate cancer index lesion by real-time elastography: considerations for focal therapy of prostate cancer. World J Urol.

[12] Rud E, Klotz D, Rennesund K (2014). Detection of the index tumour and tumour volume in prostate cancer using T2-weighted and diffusion-weighted magnetic resonance imaging (MRI) alone. BJU Int.

[13] Nygard Y, Haukaas SA, Halvorsen OJ (2014). A positive real-time elastography is an independent marker for detection of high-risk prostate cancers in the primary biopsy setting. BJU Int.

[14] Salomon G, Drews N, Autier P (2014). Incremental detection rate of prostate cancer by real-time elastography targeted biopsies in combination with a conventional 10-core biopsy in 1024 consecutive patients. BJU Int.

[15] Filson CP, Natarajan S, Margolis DJ, et al. Prostate cancer detection with magnetic resonance-ultrasound fusion biopsy: The role of systematic and targeted biopsies Cancer 2016 10.1002/cncr.29874 PMC4777653 26749141

[16] Nygard Y, Haukaas SA, Waage JE (2013). Combination of real-time elastography and urine prostate cancer gene 3 (PCA3) detects more than 97% of significant prostate cancers. Scand J Urol.

[17] Brock M, Loppenberg B, Roghmann F (2015). Impact of real-time elastography on magnetic resonance imaging/ultrasound fusion guided biopsy in patients with prior negative prostate biopsies. J Urol.

[18] Porpiglia F, Russo F, Manfredi M (2014). The roles of multiparametric magnetic resonance imaging, PCA3 and prostate health index-which is the best predictor of prostate cancer after a negative biopsy?. J Urol.

[19] Wei JT, Feng Z, Partin AW (2014). Can urinary PCA3 supplement PSA in the early detection of prostate cancer?. J Clin Oncol.

[20] Ruffion A, Devonec M, Champetier D (2013). PCA3 and PCA3-based nomograms improve diagnostic accuracy in patients undergoing first prostate biopsy. Int J Mol Sci.

[21] Aigner F, Pallwein L, Junker D (2010). Value of real-time elastography targeted biopsy for prostate cancer detection in men with prostate specific antigen 1.25 ng/ml or greater and 4.00 ng/ml or less. J Urol.

[22] Brock M, von Bodman C, Palisaar RJ (2012). The impact of real-time elastography guiding a systematic prostate biopsy to improve cancer detection rate: a prospective study of 353 patients. J Urol.

[23] Heinzelbecker J, Weiss C, Pelzer AE A learning curve assessment of real-time sonoelastography of the prostate World J Urol 2012 10.1007/s00345-012-0897-y 22828663

